# Evaluation of nutrients in bread: a systematic review

**DOI:** 10.1186/s41043-022-00329-3

**Published:** 2022-11-14

**Authors:** Zahra Aghalari, Hans-Uwe Dahms, Mika Sillanpää

**Affiliations:** 1 Environmental Health Engineer, Faculty of Public Health, Babol University of Medical Sciences, Babol, Islamic Republic of Iran; 2 Department of Biomedical Science and Environment Biology, College of Life Science, Kaohsiung Medical University, Kaohsiung, Taiwan; 3 Research Center for Environmental Medicine, KMU - Kaohsiung Medical University, Kaohsiung, 80708 Taiwan; 4 Department of Biological and Chemical Engineering, Aarhus University, Nørrebrogade 44, 8000 Aarhus C, Denmark; 5 Faculty of Science and Technology, School of Applied Physics, University Kebangsaan Malaysia, 43600 Bangi, Selangor Malaysia; 6 School of Chemistry, Shoolini University, Solan, Himachal Pradesh 173229 India; 7 Department of Marine Biotechnology and Resources, National Sun-Yat-Sen University, Kaohsiung, 80424 Taiwan

**Keywords:** Nutrients, Bread, Food hygiene, Environmental health, Nutrition

## Abstract

**Background:**

A balanced and optimized amount of nutrients in bread, which is the main food in many countries, is necessary to maintain human health. Considering the importance of nutritional values of bread in the food basket of Iranian households, the purpose of this study was to determine the nutrients and their concentrations in breads consumed in Iran.

**Methods:**

This systematic review study was performed to determine the types of nutrients in breads consumed in Iran by searching reputable international databases including Scopus and Google scholar, PubMed, Science direct, ISI (Web of Science). Data were collected according to inclusion and exclusion criteria and by searching for relevant keywords, emphasizing the types of nutrients in breads consumed in Iran. Qualitative data were collected using the standard PRISMA checklist (preferential reporting items for systematic reviews and meta-analysis). After verifying the quality of the articles, the information was entered into a checklist such as the name of the first author and year of publication of the research, type of study, number of samples, type of nutrition, type of bread and amount of nutrition measured.

**Results:**

After reviewing the information and quality of articles, 10 articles were qualified for systematic review. The review of the articles showed that different breads were experimented, including: Sangak, Barbari, Taftoon, Lavash, French and local bread. The highest number of experimented bread samples was Sangak. Examination of the articles showed that 6 nutrients were experimented in different breads such as Fe, K, Mg, Ca, Cu and Zn. The highest number of experimented in breads was related to the amount of Zn (13 times) and Cu (10 times), respectively. The results of quality assessment of articles showed that most of the studies were of good quality. The results of articles on the amount of nutrients measured in different breads showed that only in two articles the amount of nutrients was reported to be desirable. In most articles, the amount of nutrients in breads was reported to be lower or higher than standard.

**Conclusion:**

The results of this study showed that the concentration of nutrients in most articles was undesirable. It is suggested that optimal methods of enrichment of breads and flours be done with interdisciplinary cooperation between food hygiene, environmental health, nutrition, farmers and bakers. It is recommended that food hygiene and environmental health researchers investigate other nutrients (including phosphorus, selenium, manganese, boron and molybdenum) in breads and other staple foods used by people to constructive and practical measures to increase public health.

## Introduction

**Fig. 1:**
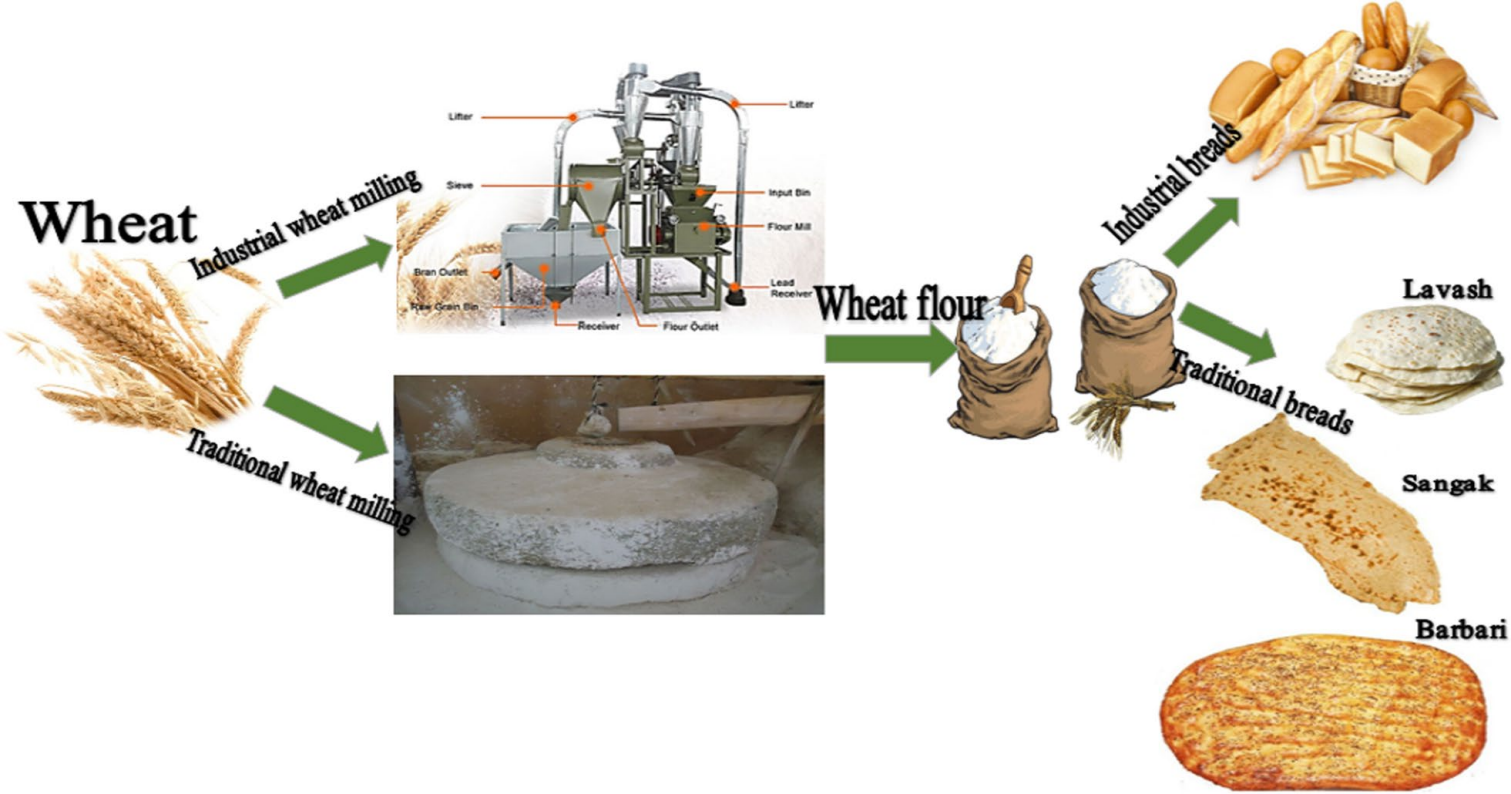
Industrial and traditional bread preparation steps

Cereals such as wheat, rice, corn and barley are the basis of human nutrition and life and provide 70% of people’s food [1, 2]. Bread contains a type of cereals called wheat, which is why bread is a staple food in developing countries, especially in Africa and Asia. Baking bread from farm to bakery has several steps (Fig. 1), and each of these steps must comply with the principles of hygiene and food quality because the steps of bread preparation affect the quality of bread. In general, there are three types of bread serial/dough sources worldwide, which are wheat bread (with gluten), bread without gluten and combined bread [3, 4].

Bread provides several nutrients such as sugar, protein, iron, calcium and a variety of vitamins [5]. An average daily intake of 300 g of bread can provide the nutrients needed by the body and create a desired nutritional status [6]. Bread can provide 1.2% of protein, 60% of thiamine and niacin, 40% of calcium and 80% of the daily iron needed by an adult [7, 8].

Deficiency of nutrients endangers human health. According to the World Health Organization, malnutrition due to nutrient deficiency is a major problem and affects more than two billion people worldwide [9]. The amount of nutrients (P, K, Se, Mg, Ca, B, MO, Cu, Zn, Mn) varies in food, including different bread samples, and each of the nutrients has a specific structure (Table 1).

**Table 1:**
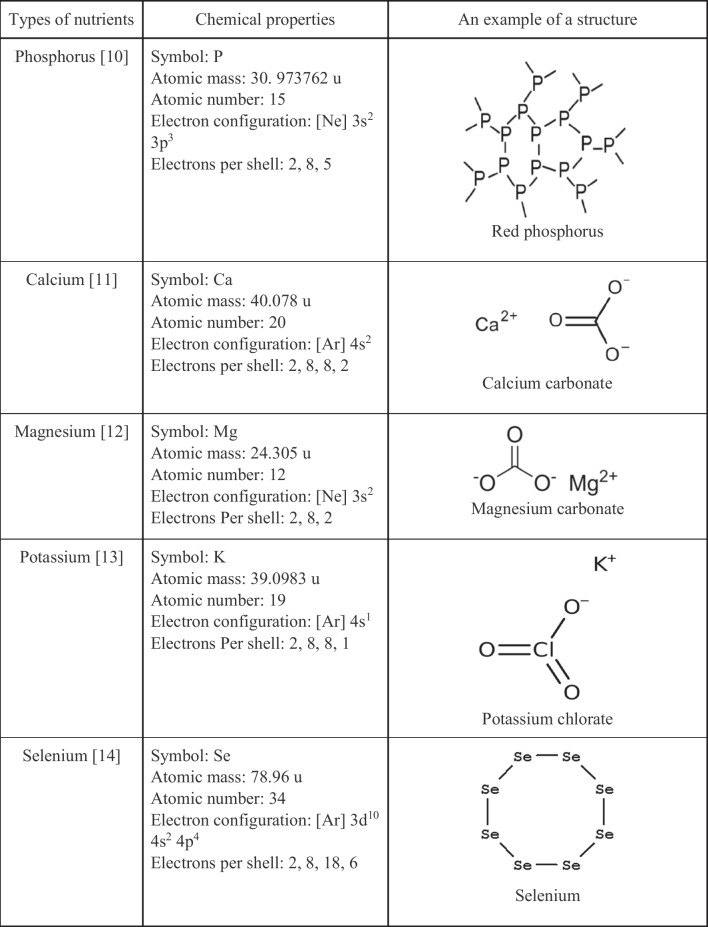

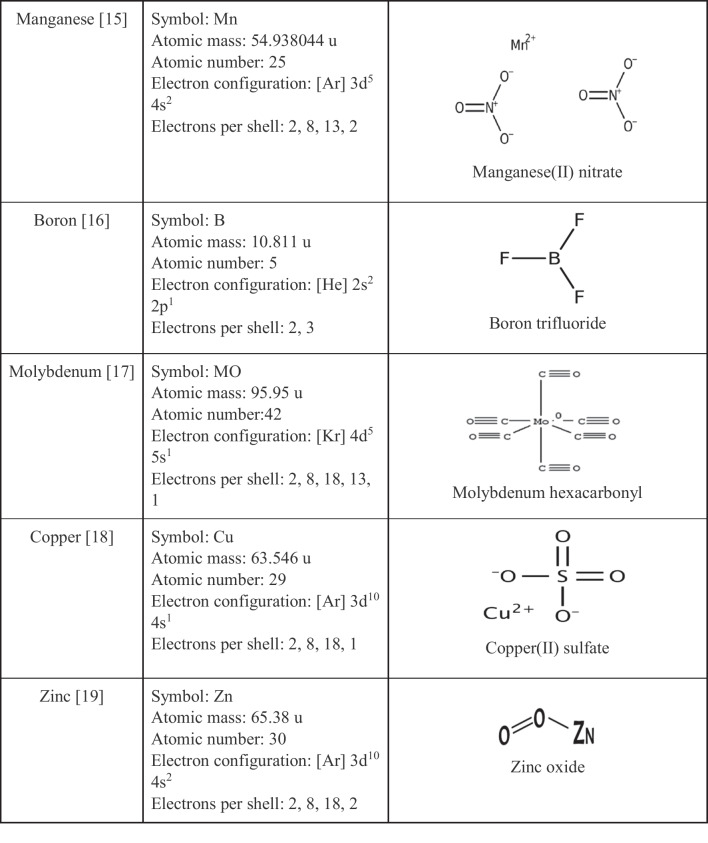
Chemical properties and structure of some nutrients

Nutrients have different biological effects on the body. Iron is one of the nutrients which deficiency causes the most common nutritional problems. For example, iron deficiency anemia is the most common type of anemia in the world [20, 21]. Zn is another nutrient that is the second most abundant trace element in the body after iron and participates in the synthesis of brain enzymes [22]. The recommended amount of Zn for daily absorption is 60 mg [23]. Zn is contained in several enzymes including carbonic anhydrase, dehydrogenase, proteinase and peptidase, and Zn in wheat reduces the carbohydrate content of leaves and stems [24]. Zn deficiency causes diseases such as impaired physical development and the immune system, reduced ability to learn, increased risk of infection and cancer [25, 26]. Cu is another nutrient that is a vital component of the body. Cu is involved in the structure, function and activity of many enzymes, in the function and activity of catalase, peroxidase and glutathione in the body. Cu deficiency increases the sensitivity of lipoproteins to peroxidation [27–29]. Se is another nutrient that prevents toxin transmission from mother to fetus. Se is an antioxidant in the body. Scientists have reported that Se is a factor that reduces aging [30].

Overcoming nutrient deficiencies is a major challenge for humans. There have been many studies on nutrients. A study by Jawad et al. was aiming at the study of copper and iron in wheat, flour and bread grains in Iraq [31]. In a study by Harmankaya et al., the concentrations of copper, iron, manganese and zinc in bread and wheat produced in Turkey were investigated [32]. In a study by Kirchmann et al., the amount of copper and iron in wheat grains in Switzerland was investigated [33].

Bread as the dominant food has a major share in the consumption pattern of households. The per capita consumption of wheat and bread in Iran is about 24 and 300 kg, respectively [34, 35]. Considering the nutritional value of bread in the food basket of Iranian households, the purpose of this study was to determine the concentration of nutrients in breads consumed in Iran.

## Methods

### Study protocol

This systematic review study was performed to determine the types of nutrients in breads consumed in Iran by searching reputable international databases including Scopus and Google scholar, PubMed, Science direct, ISI (Web of Science). Search time was from August 1 to September 10, 2020. This search was performed by two authors of this article. To ensure the receipt of all articles related to the objectives of the research, the reference of the articles was reviewed.

### Search strategy

Inquired information was collected by searching for keywords on the desired sites. Key words included: ‘Nutrients’ AND ‘Phosphorus’ OR ‘P’ AND ‘Calcium’ OR ‘Ca’ AND ‘Magnesium’ OR ‘Mg’ AND ‘Potassium’ OR ‘K’ AND ‘Selenium’ OR ‘Se’ AND ‘Manganese’ OR ‘Mn’ AND ‘Boron’ OR ‘B’ AND ‘Molybdenum’ OR ‘Mo’ AND ‘Copper’ OR ‘Cu’ AND ‘Zinc’ OR ‘Zn’ AND ‘Iron’ OR ‘Fe’ AND ‘Bread’ AND ‘Iran.’

### Inclusion criteria

Inclusion criteria for this study included several items: the year of publication, the type of nutrients, all English and Persian articles with full text that were done in Iran.

### Exclusion criteria

Criteria for excluding articles from this study included several items: lack of access to the full article, inconsistency of the subject, lack of methodology, review studies and letter to the editor, case reports, duplicate report results in other articles.

### Quality assessment

The quality of the articles was assessed based on the standard checklist PRISMA (Preferred Reporting Items for Systematic Reviews and Meta-analyses). The National Institutes of Health’s Quality Assessment Tool for Observational Cohort and Cross-Sectional Studies was used to assess the quality of the studies [36]. This checklist examines various aspects in articles such as study objectives, determining the appropriate sample size, type of study, sampling method, research community, data collection method, definition of variables and data collection tools, statistical tests, presentation of findings and results. In this study, the quality of each article was evaluated according to the checklist and scores of each article were given based on Yes, No, cannot determine not applicable and not reported (Table 2).

**Table 2:** Checklist of quality assessment tools for observational cohort and cross-sectional studies (Ref. 36)

Criteria
1. Was the research question or objective in this paper clearly stated?
2. Was the study population clearly specified and defined?
3. Was the participation rate of eligible persons at least 50%?
4. Were all the subjects selected or recruited from the same or similar populations (including the same time period)? Were inclusion and exclusion criteria for being in the study prespecified and applied uniformly to all participants?
5. Was a sample size justification, power description or variance and effect estimates provided?
6. For the analyses in this paper, were the exposure(s) of interest measured prior to the outcome(s) being measured?
7. Was the timeframe sufficient so that one could reasonably expect to see an association between exposure and outcome if it existed?
8. For exposures that can vary in amount or level, did the study examine different levels of the exposure as related to the outcome (e.g., categories of exposure, or exposure measured as continuous variable)?
9. Were the exposure measures (independent variables) clearly defined, valid, reliable and implemented consistently across all study participants?
10. Was the exposure(s) assessed more than once over time?
11. Were the outcome measures (dependent variables) clearly defined, valid, reliable and implemented consistently across all study participants?
12. Were the outcome assessors blinded to the exposure status of participants?
13. Was loss to follow-up after baseline 20% or less?
14. Were key potential confounding variables measured and adjusted statistically for their impact on the relationship between exposure(s) and outcome(s)?

### Information extraction

To extract information, all articles were reviewed according to entry and exit criteria by two independent reviewers. Both reviewers eventually summarized the information and used the views of the third author in cases where the information was inconsistent. In case of quality approval of the articles, the information extracted from the articles was entered in the checklist [37–39]. The checklist included the name of the first author and year of publication of the research, type of study, number of samples, type of nutrition, type of bread and amount of nutrition.

## Results

### Search results

**Fig. 2:**
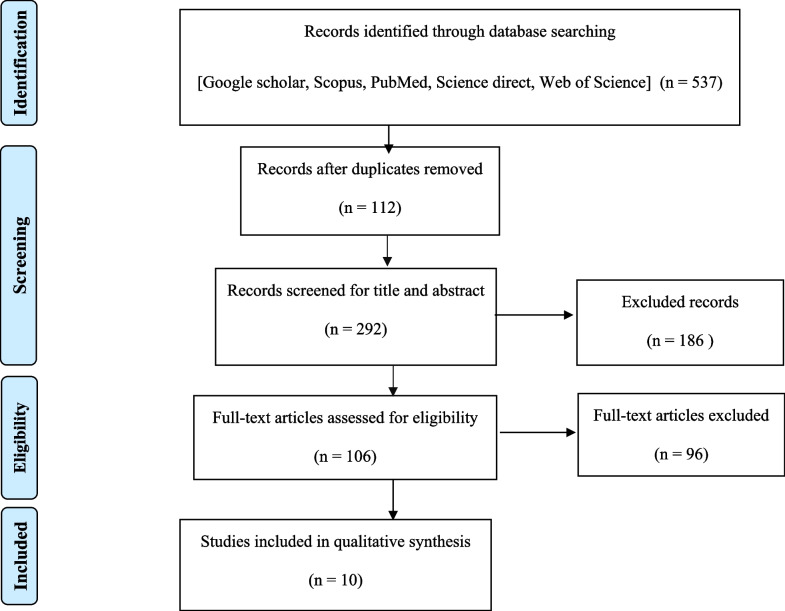
Flowchart showing the process of study selection

The steps for selecting articles are shown in Fig. 1. Using the listed keywords in combination or alone, 537 articles were found. After deleting irrelevant and duplicate articles and deleting articles based on the inclusion and exclusion criteria of this study, 10 articles remained. After reviewing the information and quality of articles, 10 articles qualified for systematic review (Fig. 2).

### Descriptive results of studies

**Fig. 3:**
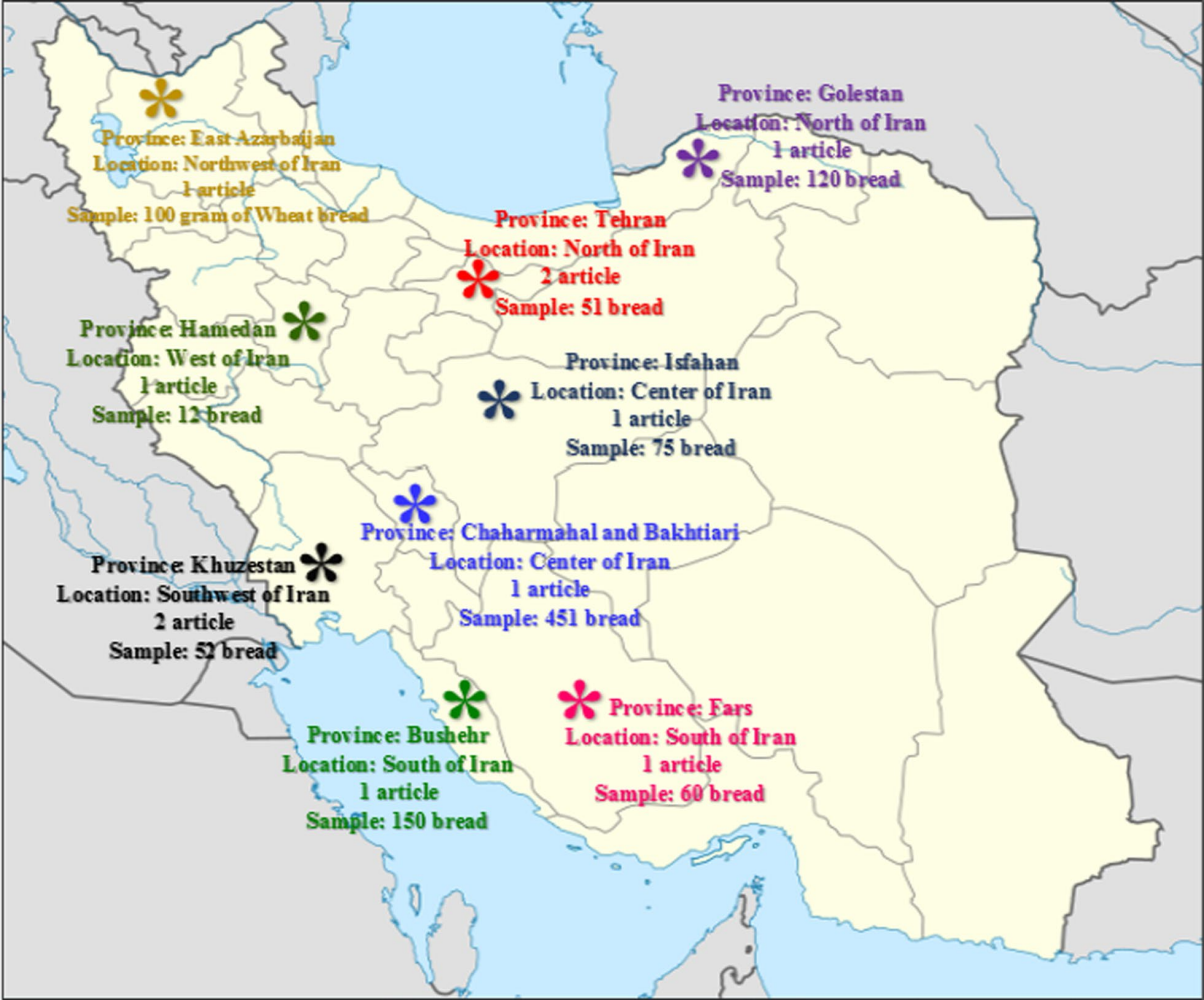
Locations of studies in 10 articles. *The locations of the provinces were shown on the map

In terms of publication time, 10 articles were published between 2004 and 2017. The largest number of articles (80%) were published between 2012 and 2017. The cities that performed nutrients on bread samples were 9 provinces from the north, south, center and west of Iran, which were shown separately on the map (Fig. 3).

**Fig. 4:**
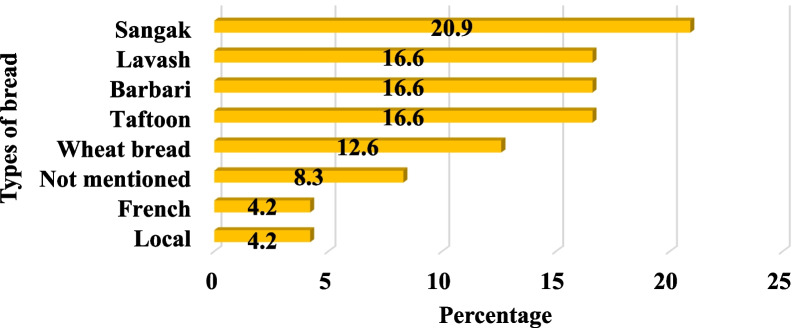
Types of bread sampled in 10 articles

The review of the articles showed that different bread samples were taken as samples, including: Sangak, Barbari, Taftoon, Lavash, French and local bread. The highest number of bread samples came from Sangak (5 articles) (Fig. 4).

**Fig. 5:**
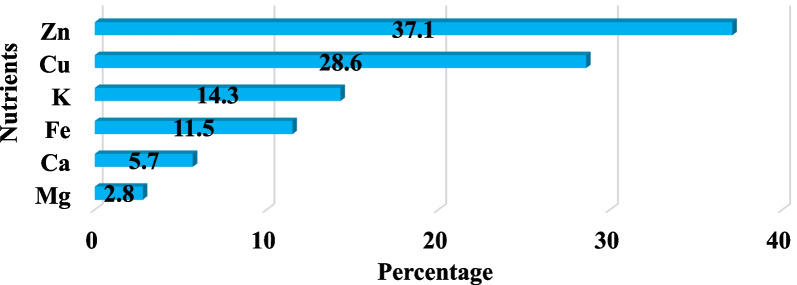
Types of nutrients measured in bread samples in 10 articles

The review of the articles showed that 6 types of nutrients (Fe, K, Mg, Ca, Cu and Zn) were experimented in different breads. The highest number of experimented in breads was related to the amount of Zn (13 times) and Cu (10 times) (Fig. 5).

### Quality assessment of studies

The results of quality assessment showed that most of the studies were of good quality. In a number of studies such as Torchi et al. [45] and Kianpoor et al. [47], the method of determining the sample size was not clear (Q5).

In the articles, participation rate of eligible persons, inclusion and exclusion criteria, the exposure(s) of interest measured prior to the outcome(s), the timeframe sufficient, exposure(s) assessed more than once over time, blinded to the exposure status of participants was not relevant and not applicable (Q3, Q4, Q6, Q7, Q10 and Q12) (Table 3).

**Table 3:** Quality of studies using NIH’s quality assessment for cohort and cross-sectional studies

References	Q1	Q2	Q3	Q4	Q5	Q6	Q7	Q8	Q9	Q10	Q11	Q12	Q13	Q14
Falahi. et al. [40]	✓	✓	NA	NA	✓	NA	NA	✓	✓	NA	✓	NA	✓	×
Sadighi et al. [41]	✓	✓	NA	NA	✓	NA	NA	✓	✓	NA	✓	NA	✓	✓
Shockravi et al. [42]	✓	✓	NA	NA	✓	NA	NA	✓	✓	NA	✓	NA	✓	✓
Badii et al. [43]	✓	✓	NA	NA	✓	NA	NA	✓	✓	NA	✓	NA	✓	✓
Hojati et al. [44]	✓	✓	NA	NA	✓	NA	NA	✓	✓	NA	✓	NA	✓	✓
Torchi et al. [45]	✓	✓	NA	NA	×	NA	NA	✓	✓	NA	✓	NA	✓	✓
Heidari et al. [46]	✓	✓	NA	NA	✓	NA	NA	✓	✓	NA	✓	NA	✓	✓
Kianpoor et al. [47]	✓	✓	NA	NA	×	NA	NA	✓	✓	NA	✓	NA	✓	✓
Tabibian et al. [48]	✓	✓	NA	NA	✓	NA	NA	✓	✓	NA	✓	NA	✓	✓
Shiralipour et al. [49]	✓	✓	NA	NA	✓	NA	NA	✓	✓	NA	✓	NA	✓	×

*Cases that were followed in the articles were marked [✓] and those that were not followed were marked [×]. Items that were not executable were also identified by the phrase not applicable [NA]

### Article features

All articles reviewed were original articles based on laboratory analysis. The highest sample size in a study by Tabibian et al. [48] [48 bread samples] and Chaharmahal and Bakhtiari [451 bread samples]. In most articles, Zn nutrients were experimented in breads such as by Falahi et al. [40], Shockravi et al. [42], Badii et al. [43], Hojati et al. [44], Heidari et al. [46], Kianpoor et al. [47] and Shiralipour [49] (Table 4).

**Table 4:** Information from articles on the amount of nutrients in Iranian breads

References	Sample size	Type of samples/City or province		Types of nutrients	Reported value		Results reported in studies
Falahi. et al. [40]	100 gr	Not mentioned/East Azerbaijan	Wheat bread	Ca	40 ± 20 mg/100 g		No analysis on the amount of nutrients
				Zn	1.79 ± 0.01 mg/100 g		
				Fe	3.9 ± 0.04 mg/100 g		
				Mg	90 ± 30 mg/100 g		
				Cu	0.38 ± 0.05 mg/100 g		
Sadighi et al. [41]	150 Bred	Not mentioned/Bushehr	Not mentioned	Fe	21.3%		Lower than standard/undesirable
					11.4%		Accepted
					66%		Good
					0		Higher than standard/undesirable
					1.3%		Lacking/undesirable
	120 Bred	Not mentioned/Golestan	Not mentioned	Fe	24.1%		Lower than standard/undesirable
					10%		Accepted
					51.7%		Good
					9.2%		Higher than standard/undesirable
					5%		Lacking/undesirable
Shockravi et al. [42]	1 Bred	Traditional bread/Tehran	Sangak	Zn	1.66 mg/100 g		Lower than standard/undesirable
				Ca	80.05 mg/100 g		
Badii et al. [43]	75 Bred	Traditional bread/Isfahan	Taftoon	Zn	1.35 ± 0.02 mg/100 g		Control
					10.10 ± 0.08 mg/100 g		High-zinc/undesirable
					5.72 ± 0.05 mg/100 g		Low-zinc/undesirable
Hojati et al. [44]	12 Bred	Traditional bread/Ahvaz	Lavash	Zn	More than 0.6 mg/100 g	Mean: 0.591 mg/100 g	Lower than standard/undesirable
			Barbari	Zn	More than 0.5 mg/100 g		
			Taftoon	Zn	More than 0.6 mg/100 g		
			Sangak	Zn	More than 0.5 mg/100 g		
Torchi et al. [45]	50 Bred	Traditional bread/Tehran	Lavash	Cu	2.805 ppm		Lower than standard/undesirable
			Barbari	Cu	2.18 ppm		
			Taftoon	Cu	2.89 ppm		
			Sangak	Cu	3.445 ppm		
Heidari et al. [46]	60 Bred	Industrial and traditional bread/Shiraz	Wheat bread	Zn	1.3 mg/kg		Lower than standard/undesirable
Kianpoor et al. [47]	12 Bred	Traditional bread/Hamedan	Lavash	Zn	5.61 ± 0.32 mg/kg		Lower than standard/undesirable
				Cu	1.66 ± 0.05 mg/kg		
			Barbari	Zn	8.84 ± 0.30 mg/kg		
				Cu	1.71 ± 0.03 mg/kg		
			Sangak	Zn	4.35 ± 0.16 mg/kg		
				Cu	1.47 ± 0.05 mg/kg		
		Industrial bread	French	Zn	3.07 ± 0.09 mg/kg		
				Cu	1.43 ± 0.01 mg/kg		
Tabibian et al. [48]	451 Bred	Traditional bread/Chaharmahal and Bakhtiari	Taftoon	K	106 ± 18 mg/100 g		Higher than standard/undesirable
			Lavash	K	103 ± 11 mg/100 g		
			Local	K	109 ± 15 mg/100 g		
			Barbari	K	112 ± 14 mg/100 g		
			Sangak	K	110 ± 10 mg/100 g		
Shiralipour et al. [49]	40 Bred	Not mentioned/Ahvaz	Wheat bread	Zn	14.08 mg/kg		No analysis on the amount of nutrients
				Cu	6.62 mg/kg		
				Fe	105.65 mg/kg		

In most articles, the type of bread experimented was Sangak such as by Shockravi et al. [42], Hojati et al. [44], Torchi et al. [45], Kianpoor et al. [47] and Tabibian et al. [48] (Table 4).

A review of article results showed that only in two articles Sadighi et al. [41] Fe level and in Badii et al. [43] Zn levels were desirable. In a study by Sadighi et al. [41], the amount of Fe in different breads was divided into 5 categories, which showed that 77.4% of the bread sampled from Bushehr and 61.7% of the bread sampled from Golestan were acceptable. In 6 articles, the amount of nutrients was reported as lower than standard, and in 2 articles, Sadighi et al. [41] and Tabibian et al. [48], the amount of nutrients was reported as higher than standard (Table 4).

## Discussion

The findings of this article showed that among the various bread (Sangak, Barbari, Taftoon, Lavash, French and local breads) the highest number of bread samples analyzed was from Sangak, for example, in the studies of Shockravi et al. [42], Hojati et al. [44], Torchi et al. [45], Kianpoor et al. [47] and Tabibian et al. [48] (Table 4). Most researchers used Sangak bread to study the characteristics of Iranian breads because the use of Sangak is recommended by Iranian nutritionists. The reason is that wholemeal flour is used to bake Sangak and wholemeal flour, and its vitamins and minerals are preserved. Sangak has high levels of vitamins, calcium, protein and iron and is easy to digest due to its high fiber content. Sangak has high levels of vitamins, calcium, protein and iron, and Sangak is easy to digest due to its high fiber content. One of the good features of Sangak is its taste, aroma, nutrition and satiety. The traditional method of baking Sangak, its shape and taste is different from other breads that are made and are highly valued in Iranian culture; especially for breakfast, the presence of Sangak is a priority [50, 51].

According to the findings of this article, researchers paid most attention to measuring the amount of Zn in breads. In a study by Falahi et al. [40] zinc level 1.79 ± 0.01 mg/ 100 g, Shockravi et al. [42] zinc level 1.66 mg/100 g, Hojati et al. [44] zinc level 0.591 mg/100 g, Heidari et al. [46] zinc level 1.3 mg/kg, Kianpoor et al. [47], the amount of zinc in Lavash, Barbari, Sangak, French breads, respectively, is 5.61 ± 0.32 mg/kg, 8.84 ± 0.30 mg/kg, 4.35 ± 0.16 mg/kg and 3.07 ± 0.09 mg/kg. In the analysis of the results of all studies, it was mentioned that the amount of zinc was less than the standard. Zinc deficiency in bread can cause serious problems for the health of the Iranian people because bread is one of the main sources of zinc in Iranian food. One of the reasons for zinc deficiency in Iran is insufficient intake of zinc from the diet, high consumption of grains, especially unfermented bread [52, 53]. Various studies have reported that zinc deficiency in the Middle East is due to a poor diet and zinc deficiency is common in developing countries such as Iran [54, 55]. Since deficiency causes problems such as hair loss, imbalance when walking in the elderly, anorexia, taste disturbance, lethargy, fainting, behavioral disorders and growth retardation in children [56, 57], attention should be paid to flour fortification and consumer breads in Iran.

Based on the findings of this article, it was reported about K in Tabibian et al. [48] K level, lower than standard and undesirable. Low levels of potassium in bread can cause problems over time. Potassium disorders are the most common electrolyte abnormality detected in clinical practice [58]. Potassium deficiency causes fatigue, drowsiness, muscle weakness, constipation, irregular heartbeat and delayed gastric emptying. Many studies have shown that potassium plays an essential role in the normal functioning of cells, and a diet with adequate potassium levels is important for the prevention of cancer and cardiovascular disease [59, 60]. Since potassium is one of the basic elements of the body that the body needs the desired amount for a wide range of functions, it is necessary to examine the amount of potassium in people’s basic foods, including bread, and if potassium is undesirable, health and nutritional measures should be taken.

One of the strengths of this study is addressing an issue related to food quality, namely the amount of nutrients in bread consumed by people. Although bread is one of the main foods of Iranian people, researchers did less research on it. Another strength of this study was the review of articles without time limit so that all related articles were included in this study.

## Conclusions

According to the findings of this study and by comparing the amount of different nutrients (Fe, K, Mg, Ca, Cu, Zn) in bread, it was determined that the amount of nutrients in breads sampled in an unfavorable condition is lower than standard or higher than standard. It is suggested that optimal methods of enrichment of breads and flours should be done with interdisciplinary cooperation between food hygiene, environmental health, nutrition, farmers and bakers. It is recommended that food hygiene and environmental health researchers investigate other nutrients (including phosphorus, selenium, manganese, boron and molybdenum) in breads and other staple foods used by people as constructive and practical measures to increase public health.

## Data Availability

The datasets used and analyzed during the current study are available from the corresponding author on reasonable request.

## Abbreviations

P: Phosphorus
Ca: Calcium
Mg: Magnesium
K: Potassium
Se: Selenium
Mn: Manganese
B: Boron
Mo: Molybdenum
Cu: Copper
Zn: Zinc
WHO: World Health Organization
ISI: Web of Science
PRISMA: Preferred Reporting Items for Systematic Reviews and Meta-analyses
